# *NR5A1*/SF-1 Collaborates with Inhibin α and the Androgen Receptor

**DOI:** 10.3390/ijms251810109

**Published:** 2024-09-20

**Authors:** Rawda Naamneh Elzenaty, Chrysanthi Kouri, Idoia Martinez de Lapiscina, Kay-Sara Sauter, Francisca Moreno, Núria Camats-Tarruella, Christa E. Flück

**Affiliations:** 1 Pediatric Endocrinology, Diabetology and Metabolism, Department of Pediatrics, Inselspital, Bern University Hospital, University of Bern, 3010 Bern, Switzerland; rawda.naamneh@students.unibe.ch (R.N.E.); chrysanthi.kouri@students.unibe.ch (C.K.); idoia.martinezdelapiscina@unibe.ch (I.M.d.L.); kay.sauter@unibe.ch (K.-S.S.); 2 Department of BioMedical Research, University of Bern, 3008 Bern, Switzerland; 3 Graduate School for Cellular and Biomedical Sciences, University of Bern, 3012 Bern, Switzerland; 4 Biobizkaia Health Research Institute, Cruces University Hospital, University of the Basque, 48903 Barakaldo, Spain; 5 CIBER de Diabetes y Enfermedades Metabólicas Asociadas (CIBERDEM), Instituto de Salud Carlos III, 28029 Madrid, Spain; 6 CIBER de Enfermedades Raras (CIBERER), Instituto de Salud Carlos III, 28029 Madrid, Spain; 7 Endo-ERN, 1081 HV Amsterdam, The Netherlands; 8 Department of Pediatrics, Hospital Infantil La Fe, 46026 Valencia, Spain; moreno_framac@gva.es; 9 Growth and Development Research Group, Vall d’Hebron Research Institute, 08035 Barcelona, Spain; nucata@yahoo.es

**Keywords:** steroidogenic factor 1 (SF-1/*NR5A1*), inhibin α, androgen receptor, disorders of sex development (DSD), 46,XY DSD, hypospadias, oligogenicity

## Abstract

Steroidogenic factor 1 (SF-1) is a nuclear receptor that regulates steroidogenesis and reproductive development. *NR5A1*/SF-1 variants are associated with a broad spectrum of phenotypes across individuals with disorders of sex development (DSDs). Oligogenic inheritance has been suggested as an explanation. SF-1 interacts with numerous partners. Here, we investigated a constellation of gene variants identified in a 46,XY severely undervirilized individual carrying an ACMG-categorized ‘pathogenic’ *NR5A1*/SF-1 variant in comparison to the healthy carrier father. Candidate genes were revealed by whole exome sequencing, and pathogenicity was predicted by different in silico tools. We found variants in *NR1H2* and *INHA* associated with steroidogenesis, sex development, and reproduction. The identified variants were tested in cell models. Novel SF-1 and NR1H2 binding sites in the *AR* and *INHA* gene promoters were found. Transactivation studies showed that wild-type *NR5A1*/SF-1 regulates *INHA* and *AR* gene expression, while the *NR5A1*/SF-1 variant had decreased transcriptional activity. NR1H2 was found to regulate *AR* gene transcription; however, the NR1H2 variant showed normal activity. This study expands the *NR5A1*/SF-1 network of interacting partners, while not solving the exact interplay of different variants that might be involved in revealing the observed DSD phenotype. It also illustrates that understanding complex genetics in DSDs is challenging.

## 1. Introduction

Steroidogenic factor 1 (*NR5A1*/SF-1) is a nuclear receptor and a master regulator of steroidogenesis and reproductive development. *NR5A1*/SF-1 controls several steps of gonadal and adrenal development [1,2]. Therefore, the disruption of *NR5A1*/SF-1 may lead to abnormalities in steroidogenic and reproductive tissues. *Nr5a1*/Sf-1 knockout mice have a sex reversal phenotype and adrenocortical insufficiency, while heterozygous *Nr5a1*/Sf-1 mice exhibit hypoplasia of the adrenal glands and testes [3,4]. Human genetic variants in *NR5A1*/SF-1 may lead to disorders/differences of sex development (DSDs) associated with a wide range of phenotypes, and very few individuals with *NR5A1*/SF-1 variants show an adrenal phenotype. Mice models do not recapitulate the broad phenotype seen in humans [5,6,7]. *NR5A1*/SF-1 variants are mostly found in a heterozygous state and are scattered throughout the whole gene without any obvious hotspots [5,6,7]. To assess the pathogenicity of the identified *NR5A1*/SF-1 variants, numerous in vitro studies showed mixed results concerning confirmation of the disease-causing mechanism as required by the current guidelines of the American College of Medical Genetics and Genomics (ACMG) [6,8].

*NR5A1*/SF-1 has a wide network of interactions, including many transcription factors, co-modulators, posttranslational modulators, and signaling molecules [1]. Therefore, it was suggested that the broad phenotypes among patients with DSD may be explained by oligogenic inheritance, where multiple genetic variants together with *NR5A1*/SF-1 might contribute to a specific DSD phenotype of an individual [5,6,9,10,11,12,13,14]. Oligogenic causation has been reported for other endocrine disorders, for instance congenital hypogonadotropic hypogonadism or congenital hypothyroidism [15,16,17,18]. In both, a synergistic or collaborative role of different gene variants was assumed probable [15,16,17,18]. Similarly, *NR5A1*/SF-1 variants, in combination with other variants in DSD-related genes, were identified in several individuals using next-generation sequencing (NGS) methods [6,9,10,12,13,14,19,20,21,22]. However, a mechanistic confirmation of oligogenicity in DSDs related to *NR5A1*/SF-1 is missing.

Bioinformatic tools for testing combinatory variants are beneficial for identifying potential oligogenicity but are scarce [10,23,24,25]. Moreover, the contribution of the predicted variants needs to be confirmed experimentally by in vitro or ex vivo studies [26]. The activity of *NR5A1*/SF-1 as a transcription factor has classically been analyzed in cell models by testing its transactivation activity on the promoter constructs of targeted genes and by nuclear translocation studies [6]. These studies have enhanced our understanding of the effect of *NR5A1*/SF-1 on specific target genes. Therefore, in this study, we investigated the possible mechanism of interaction of genetic variants found in a 46,XY individual with a severe DSD phenotype carrying an *NR5A1*/SF-1 mutation in comparison with his healthy carrier father using bioinformatic and in vitro, cell-based methods. We performed whole exome sequencing (WES) and a comprehensive data analysis guided by the patient’s phenotype to identify candidate variants in additional genes, which were then investigated by transactivation studies in different cell models to elucidate their interaction with *NR5A1*/SF-1 and beyond.

## 2. Results

### 2.1. Phenotypic Characterization

The patient manifested at birth with a 46,XY DSD consisting of micropenis, scrotal hypospadias, bilateral cryptorchidism, and the absence of Müllerian ducts (previously reported in [9,27]). The patient had hypospadias repair at the age of 3.8 and 4.5 years and a right and left orchidopexy at the age of 2.5 and 5.7 years, respectively. An adrenocorticotropic hormone (ACTH) stimulation test was performed at the age of 3 years and revealed a normal cortisol response. At 11 years of age, ultrasound showed testes in the scrotum (volume of 1 cm^3^ and 0.8 cm^3^). The patient had spontaneous puberty at the age of 11.8 years with normal testosterone (T) and luteinizing hormone (LH) levels, but elevated follicle-stimulating hormone (FSH) levels (38.2 mIU/mL) for the Tanner stage. Normal ACTH and cortisol levels were confirmed. A testicular biopsy was taken at the age of 12.4 years, revealing seminiferous tubules devoid of germinal cells. An anthropometric evaluation at the age of 14.8 years indicated a weight of 57.9 kg (−0.28 SDS), height of 167.2 cm (−0.25 SDS), and BMI of 20.7 kg/m2 (−0.18 SDS). Growth velocity was 9.4 cm/year (3.45 SD). The patient had a testicular volume of 6 mL/8 mL, with Tanner stage 3 for pubic hair and genital status; breast development was B1. A further biochemical evaluation was performed at the age of 15 years, presented in Table 1. A family history revealed healthy parents and was unremarkable for DSDs.

### 2.2. Genotypic Characterization

The index patient and the father carry a heterozygous c.58G>C; p.(Val20Leu) variant in the *NR5A1* gene [27]. The mother’s DNA was not available for genetic investigations. The c.58G>C; p.(Val20Leu) variant was previously classified as “pathogenic” according to the ACMG criteria and most of the in silico tools (Table 2). Because of the discrepancy in phenotype between father and son, WES was performed on both. A variant analysis was conducted using a tailored algorithm to search for the oligogenic etiology of DSDs linked to *NR5A1*/SF-1 [9]. A single heterozygous variant, c.675T>G; p.(Ser225Arg), in the *INHA* gene was found in the patient but not in the healthy father; this variant was classified as a variant of uncertain significance (VUS) [9]. Recently, the aforementioned algorithm was updated [11], and the WES data of the index patient were reanalyzed. This reanalysis revealed four additional heterozygous variants in the patient in different genes, *NR1H2*, *TCF7L2*, *NIBAN1*, and *SCUBE2* (Table 2). The *INHA* variant was re-classified as benign (B) according to the ACMG criteria [28] and in silico tools (Table 2). Three of the newly identified candidate variants were classified as VUS, while in silico tools showed variable predictions. The variant in the *TCF7L2* gene was classified as likely benign (LB) according to the ACMG and in silico tools (Table 2). Additionally, in ORVAL, the variants in the *TCF7L2* c.1535C>G; p.(Pro512Arg), *NIBAN1* c.929G>A; p.(Arg310His), and *SCUBE2* c.692C>T; p.(Thr231Ile) genes were predicted to form a pathogenic oligogenic combination with the *NR5A1* gene (Table 2).

To investigate the possible contribution of the newly identified variants to the DSD phenotype of the patient, we searched the literature for reported interactions between the different genes and *NR5A1*/SF-1 (Table 3). In addition, we searched for the phenotype associated with these variants in human and mice models (Table 3). Apart from the *NR5A1*/SF-1 gene, which is associated with a wide phenotypic spectrum of DSDs [5,6], we found that only two genes (*NR1H2* and *INHA*) were involved in steroidogenesis, sex development, and/or reproduction. Therefore, the identified variants in the three other genes were excluded from further studies due to their different biological roles (see Table 3 for more details).

### 2.3. Characterization of the Identified Variants in NR5A1/*SF-1*, NR1H2, and INHA

We conducted a conservation comparison for each of the three variants *NR5A1*/SF-1 c.58G>C; p.(Val20Leu), c.515_516insCAA; p.(Arg171_Lys172insAsn) *NR1H2*/LXRβ, and c.675T>G; p.(Ser225Arg) *INHA* against their wild-type (WT) protein (Figure S1). Comparison for variant and WT SF-1 similarity across species revealed that the variants and the surrounding regions are highly conserved (Figure S1). Similar results were found for inhibin α. The insertion of the asparagine amino acid in the LXRβ variant protein (encoded by the *NR1H2* gene) may affect two conserved amino acids across different species.

### 2.4. In Vitro Functional Testing of Selected Variants

**Figure 1:**
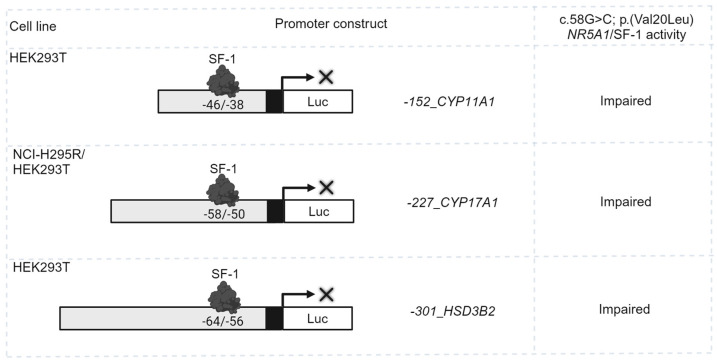
Previously reported transcriptional activity of the c.58G>C; p.(Val20Leu) *NR5A1*/SF-1 variant tested on three different steroidogenic promoter constructs in HEK293T and NCI-H295R cell lines, figure drawn based on data of [27].

The pathogenicity of the c.58G>C; p.(Val20Leu) *NR5A1*/SF-1 variant was previously assessed by activation testing on three different promoter constructs of three steroid enzymes (e.g., −*152_CYP11A1*, −*227_CYP17A1* and −*301_HSD3B2*) in HEK293T and NCI-H295R cells, revealing impaired transcriptional activation on all three gene promoters (Figure 1; data reproduced from [27]). In pursuit of explaining the DSD phenotype of the patient in comparison with the healthy carrier father for the *NR5A1*/SF-1 variant, two additional variants in the *INHA* and *NR1H2* genes were functionally studied in vitro for their possible disease-causing effect.

**Figure 2:**
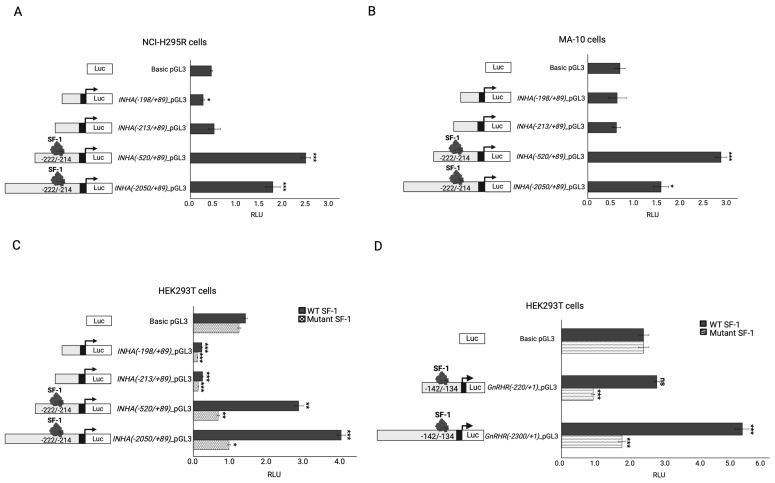
*NR5A1*/SF-1 regulates the expression of genes crucial for the function of steroidogenic tissues and the hypothalamic–pituitary–gonadal axis. (**A**,**B**) Endogenous *NR5A1*/SF-1 transcriptional activity on different *INHA* promoter–reporter constructs in steroidogenic cell lines: (**A**) adrenal NCI-H295R cells and (**B**) mouse Leydig MA-10 cells. Cells were transiently transfected only with the −*198_INHA*, −*213_INHA*, −*520_INHA*, −*2050_INHA* promoter luciferase reporter constructs. (**C**) The ability of the WT or mutant c.58G>C; p.(Val20Leu) *NR5A1*/SF-1 to activate four different promoter–reporter constructs of the *INHA* gene was tested in the non-steroidogenic HEK293T cell line. The cells were transiently co-transfected with WT or mutant c.58G>C; p.(Val20Leu) *NR5A1*/SF-1 and −*198_INHA*, −*213_INHA*, −*520_INHA*, −*2050_INHA* promoter luciferase reporter constructs. (**D**) The ability of the WT or mutant c.58G>C; p.(Val20Leu) *NR5A1*/SF-1 to activate the two different promoter–reporter constructs of the *GnRHR* gene was tested in HEK293T cells. Cells were transiently co-transfected with WT or mutant c.58G>C; p.(Val20Leu) *NR5A1*/SF-1 and −*220_GnRHR*, −*2300_GnRHR* promoter luciferase reporter constructs. In all experiments, the luciferase activity was measured with the Dual-Luciferase assay system (Promega). Results are shown as the mean ± standard error of the mean (SEM) of three to five independent experiments, all performed in duplicate. ns, not significant; *, *p* < 0.05; **, *p* < 0.01; ***, *p* < 0.001. RLU, relative light units.

The transcriptional regulation of *INHA* by *NR5A1*/SF-1 was tested by transfecting four *INHA* promoter constructs in steroidogenic adrenal NCI-H295R cells and Leydig MA-10 cells, which both express endogenous *NR5A1*/SF-1. Only the two longer constructs, −*520INHA* and −*2050INHA*, containing a consensus *NR5A1*/SF-1 binding site (*5′-TCATGGCCA-3′* at −*222*/−*214*) were activated by SF-1, while the two constructs lacking the *NR5A1*/SF-1 and/or cAMP-responsive element (*5′-TGCGTCA-3′* at −*205*/−*199*) were not (Figure 2A,B). In order to confirm that this activation was specifically achieved by *NR5A1*/SF-1, the constructs were co-transfected with WT or variant c.58G>C; p.(Val20Leu) *NR5A1*/SF-1 in non-steroidogenic HEK293T cells that do not express *NR5A1*/SF-1. Similar results were found; only the constructs −*520INHA* and −*2050INHA* were activated by the WT *NR5A1*/SF-1 (Figure 2C). However, variant c.58G>C; p.(Val20Leu) *NR5A1*/SF-1 showed an impaired activation (Figure 2C) of *INHA* promoters. Overall, these results indicate that SF-1 is a transcriptional regulator of *INHA* expression. 

As the role of *INHA* in sex development appears to be through the regulation of the hypothalamic–pituitary–gonadal (HPG) axis [37], we investigated the combined impact of *NR5A1*/SF-1, inhibin α, and activin A on *GnRHR* gene expression. WT SF-1 was found to activate the −*2300GnRHR* promoter construct harboring an *NR5A1*/SF-1 binding site at −*142*/−*134*, while mutant SF-1 showed a significantly lower activation (Figure 2D). By contrast, the addition of activin A, or the overexpression of WT *INHA*, in the absence or presence of *NR5A1*/SF-1, had no additional impact on −*2300GnRHR* promoter activation. To assess the impact of inhibin α on *NR5A1* expression, we overexpressed *INHA* in adrenal NCI-H295R cells, which express endogenous *NR5A1*. However, neither WT nor mutant inhibin α had an effect on *NR5A1* expression levels.

**Figure 3:**
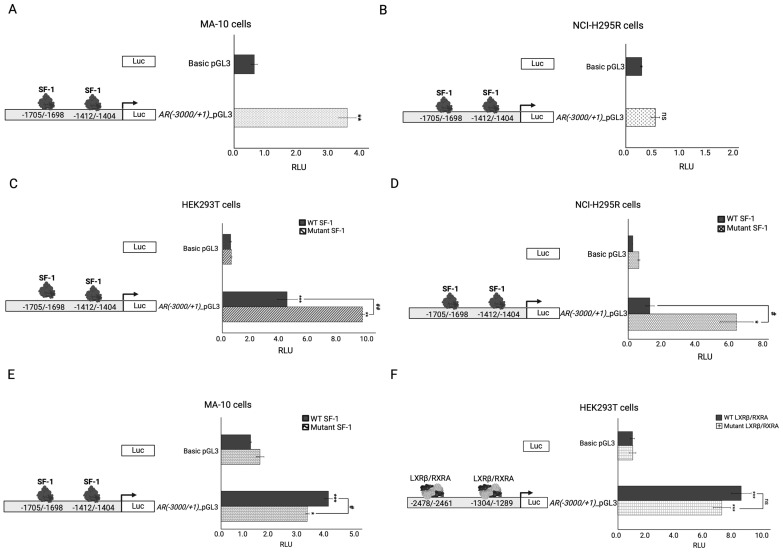
The transcriptional regulation of the *AR* in different cell lines. (**A**,**B**) The *AR* promoter construct’s transcriptional regulation was investigated in the steroidogenic cell line MA-10 (**A**,**B)** NCI-H295R. Cells were transiently transfected only with the −*3000_AR* promoter luciferase reporter construct. (**C–E**) The ability of the WT or mutant c.58G>C; p.(Val20Leu) *NR5A1*/SF-1 to activate the *AR* promoter reporter construct was tested in (**C**) HEK293T, (**D**) NCI-H295R, and (**E**) MA-10 cells. The cells were transiently co-transfected with WT or mutant c.58G>C; p.(Val20Leu) *NR5A1*/SF-1 and −*3000_AR* promoter luciferase reporter construct. (**F**) The ability of the WT or mutant c.515_516insCAA; p.(Arg171_Lys172insAsn) *NR1H2*/LXRβ and WT RXRA hetero-tetramer to activate the *AR* promoter–reporter constructs was tested in HEK293T cells. Cells were transiently co-transfected with WT or mutant c.515_516insCAA; p.(Arg171_Lys172insAsn) *NR1H2*/LXRβ, WT RXRA, and the −*3000_AR* promoter luciferase reporter construct. In all experiments, the luciferase activity was measured with the Dual-Luciferase assay system (Promega). Results are shown as the mean ± standard error of the mean (SEM) of three to five independent experiments, all performed in duplicate. RLU, relative light units. Significance of the experimental group vs. the control group: *, *p* < 0.05; **, *p* < 0.01; ***, *p* < 0.001. Significance between the experimental groups: #, *p* < 0.05; ##, *p* < 0.01.

To test the impact of the identified variants in the *NR1H2* and *NR5A1*/SF-1 genes, the androgen receptor (*AR*) was chosen as a target. The AR was reported to regulate *NR1H2*/LXβ expression [56] and to interact with *NR5A1*/SF-1 as part of the transcriptional machinery modulating the expression of specific genes (e.g., *LHB*) [57]. However, its regulation by these nuclear factors has not been reported so far. Therefore, we first tested whether the *AR* promoter is regulated by endogenous *NR5A1*/SF-1 (Figure 3A,B). The *-3000AR* promoter–reporter construct was significantly activated in steroidogenic MA-10 Leydig cells (Figure 3A); however, no activation was detected in the adrenal NCI-H295R cells (Figure 3B). The possible *NR5A1*/SF-1 binding sites in the *AR* promoter were searched manually, and we found *5′-TGACCTCT-3′* at −*1705*/−*1698* and *5′-TGGCCTCC-3′* at −*1412*/−*1404*. Interestingly, the −*3000AR* construct was found to be differentially regulated by *NR5A1*/SF-1 overexpression in three different cell lines (Figure 3C–F). WT *NR5A1*/SF-1 significantly activated the −*3000AR* construct in HEK293T and MA-10 cells, but not in NCI-H295R cells (Figure 3C–E). Surprisingly, the mutant c.58G>C; p.(Val20Leu) *NR5A1*/SF-1 activated the *AR* construct in HEK293T and NCI-H295R cells (Figure 3C,D) more than in MA-10 Leydig cells (Figure 3E). Lastly, the *AR* was tested for its transcriptional regulation by LXRβ/RXRA in HEK293T cells. Both the WT and mutant c.515_516insCAA, p.(Arg171_Lys172insAsn) LXRβ/RXRA hetero-tetramers were able to significantly activate the −*3000AR* construct, but no significant difference was found for the variant (Figure 3F).

## 3. Discussion

*NR5A1*/SF-1 variants are reported in 46,XY and 46,XX individuals presenting with a variable severity of DSDs ranging from healthy to opposite sex phenotypes. So far, a genotype–phenotype correlation has not been found [5,6]. Oligogenic inheritance could be a possible explanation for this broad phenotype, where multiple gene variants may contribute to a unique DSD phenotype for each individual [5,6,9,10,12,27,58]. SF-1 has been reported to have many interacting partners. In several DSD individuals carrying *NR5A1*/SF-1 variants, additional variants in DSD-related genes have been described (Table S1). In this study, we investigated the possible genetic interplay of several variants of a 46,XY DSD individual. By conducting a WES analysis on both the healthy carrier father and the index patient, we identified five additional gene variants in the patient only. Four of these variants had not been previously reported. According to the literature, only the *INHA* and *NR1H2* genes are involved in steroidogenesis, sex development, and/or reproduction, while the other genes are either involved in diabetes or cancer (Table 3).

*NR5A1*/SF-1 is a regulatory hub for numerous interacting partners [1]. Conducting functional assays, we were able to show that both *INHA* and *NR1H2* are part of the *NR5A1*/SF-1 interaction network. A finding that was not previously reported.

The *INHA* gene encodes the α subunit needed for the assembly of the dimeric glycoproteins termed A and B inhibins that suppress FSH secretion from the pituitary and play an important role in modulating activin levels [37]. In addition, inhibins play a role in Sertoli and Leydig cell function, spermatogenesis, and sperm count [59]. The rat inhibin α subunit can be detected at a very early stage of testicular development following the formation of the testicular cord, and it is thought to play an important autocrine/paracrine role [60]. In mice, disruption of the *Inha* gene leads to the development of gonadal sex cord–stromal tumor and infertility [61]. In contrast, the human inhibin α subunit has been detected in the fetal testis only by 16 weeks of gestation following gonadal differentiation, specifically in interstitial and Sertoli cells; it contributes to normal testicular development [60]. Biallelic *INHA* variants were found to be associated with 46,XY DSD in humans [38,40]. A homozygous 2 bp deletion c.208_209delAG, p.(R70Gfs*3) in the *INHA* gene was found in two brothers with hypospadias, hypergonadotropic hypogonadism, gynecomastia, and azoospermia [38]. Still, the specific functional role of *INHA* in male sex development and reproduction is largely unknown.

In this study, we identified a regulatory *NR5A1*/SF-1 binding site in the human *INHA* gene promoter and show that *INHA* is transcriptionally regulated by *NR5A1*/SF-1. Relating this to the investigated DSD patient, his *NR5A1*/SF-1 variant showed reduced activity on the *INHA* promoter.

Investigating the specific contribution of the c.675T>G, p.(Ser225Arg) *INHA* variant to the patient’s DSD phenotype was more challenging. The c.675T>G, p.(Ser225Arg) *INHA* variant affects a highly conserved amino acid (Figure S1) located in the αN pro-domain in the inhibin α precursor protein, which is further processed to obtain its mature and active form [62]. Very little information is available regarding the function of this region and its underlying regulatory mechanisms. However, it is predicted to contribute to the proper folding, processing, and export of inhibins (predominantly inhibin B) from Sertoli cells in the testis to the serum [62,63,64]. It has been previously reported that *Nr5a1*/Sf-1 can stimulate *Gnrhr* expression in mice and humans [65,66]. Additionally, activin A was shown to enhance *Gnrhr* expression in mice; however, its role in regulating human *GnRHR* is unknown [67]. Therefore, we explored the potential collaborative activation of the human *GnRHR* gene by activin A and *NR5A1*/SF-1 and their inhibition by inhibin α. Unfortunately, we did not observe any additional increase in *GnRHR* promoter activity by activin A. Similarly, upon the addition of the WT inhibin α, *GnRHR* promoter activity was not affected in the presence or absence of activin A. Therefore, the mechanistic proof of the contribution of the c.675T>G, p.(Ser225Arg) *INHA* variant to the phenotype found in our patient remains elusive.

Another variant identified in the patient was in the *NR1H2* gene, which encodes the liver X receptor β (LXRβ), an important modulator of lipid and cholesterol homeostasis [68]. It forms an obligate heterodimer with the retinoid X receptor (RXR) to govern gene transcription by binding to specific LXR-responsive elements [68]. The *Nr1h2* gene was found to be strongly expressed at 16.5 days postcoitum (dpc) in the mouse embryonic testis, specifically in Sertoli cells, where its expression persists into adulthood [69]. lxrβ^−/−^ knockout mice present with excessive cholesterol accumulation in Sertoli cells and dysregulated spermatogenesis, while lxrαβ^−/−^ mice present with a severe infertility phenotype [70]. Similarly, lower expression levels of *NR1H2* were detected in the testis of infertile men with azoospermia [35,36]. However, the specific function of *NR1H2* in the human developing testis has not been elucidated.

Due to the fact that both *NR1H2* and *NR5A1*/SF-1 play important roles in androgen homeostasis and male fertility [1,33,35,36,70,71,72], we tested their transcriptional activity on the *AR* promoter. Functional studies showed that *NR5A1*/SF-1 is a cell-specific transcriptional regulator of the *AR* in Leydig MA-10 cells but not in adrenal NCI-H295R cells. Overexpression of WT *NR5A1*/SF-1 enhanced the *AR* reporter activity.

Going back to our patient, we found that the mutant *NR5A1*/SF-1 was not able to activate the *AR* to the same degree as WT in MA-10 Leydig cells. By contrast, transactivation studies of the *NR5A1*/SF-1 variant in adrenal NCI-H295R and non-steroidogenic HEK293T cells revealed contradictory results, suggesting that the specific background of the Leydig cell is necessary for showing that specific interplay.

AR activity is regulated by complex mechanisms [73]. It is influenced by various transcription factors and coregulators involved in multiple cellular pathways [73,74,75]. The most recent study showed that *AR* activity is modulated by the transcription factor disheveled-associated activator of morphogenesis 2 (DAAM2), a cytoskeletal regulator of formin and actin. In vitro studies of genital skin-derived fibroblasts (GSFs) from patients with androgen insensitivity syndrome (AIS) type II and DAAM2 variants showed reduced dihydrotestosterone (DHT)-induced AR activity compared to WT GSFs [74]. Moreover, the AR is epigenetically regulated; alterations in methylated CpG regions within the proximal *AR* promoter were found to inhibit *AR* transcription in GSFs from several patients with AIS type II [75]. In our study, we show that the LXRβ/RXRA heterodimer is a transcriptional activator of the *AR*, strengthening *NR1H2*/LXRβ association with male fertility in line with previous reports [33,35,36,70]. However, the c.515_516insCAA; p.(Arg171_Lys172insAsn) *NR1H2*/LXRβ VUS had a similar transcriptional activity on the *AR* reporter as WT; thus, its contribution to the DSD phenotype is in doubt.

The proper reporting of oligogenic variant combinations requires thorough genetic testing and functional evidence of the pathogenicity of the causal variants [8,26]. Advancements in NGS methods (WES, whole genome sequencing) have significantly enhanced the yield from efforts to identify the possible genetic causes of DSDs [25], and this is especially true for gene variants that occur in combination with *NR5A1*/SF-1 variants. In fact, more than 70 different gene variants have been reported in association with *NR5A1*/SF-1 variants in individuals with DSD (Table S1) [6,9,11,12,13,14,58,76]. We performed a WES analysis on individuals with DSD and *NR5A1*/SF-1 variants as part of the SF1next study [5] and found several additional novel gene variants (unpublished data), suggesting digenic or oligogenic causation for the disease. To confirm an oligogenic disease mechanism can be difficult, as often, appropriate experimental models are missing to account for multiple genetic hits and/or the smaller effect size assumed for individual variants occurring in combination. In our 46,XY DSD index patient, five gene variants were identified, of which only the *NR5A1*/SF-1 variant was also found in the healthy father. While the variants in *TCF7L2*, *NIBAN1*, and *SCUBE2* were deemed irrelevant for the observed phenotype, the *NR1H2* and *INHA* genes were found to be interacting partners of *NR5A1*/SF-1. However, our efforts to show the disease-causing effects of the identified variants in the *NR1H2* and *INHA* genes of our index case through functional studies were not successful.

Future studies using patient-derived biomaterials may help in assessing oligogenic mechanisms. The cellular reprogramming of induced pluripotent stem cells (iPSCs) carrying the specific individual’s genetic background may inform us about the variants’ effects on steroidogenesis and sex development. Recently, in vitro systems for the differentiation of iPSCs towards gonadal progenitors and Sertoli-like cells have been established [77,78]. Rescue experiments in iPSCs originating from a 46,XY DSD patient with an *NR5A1*/SF-1 variation showed the disease mechanism on sex determination [77]. Unfortunately, even these promising models have limitations, including the availability of patients’ biological materials and the variability and difficulty in obtaining the robust maturation of fully functional iPSC-derived somatic cells (e.g., Sertoli- and Leydig-like cells). Therefore, even these experiments may not fully recapitulate the phenotype when used for disease modeling. Moreover, the challenge to rescue multiple combined variants and assess their effect on the overall phenotype remains.

In conclusion, we provide new functional evidence that *NR5A1*/SF-1 regulates the transcription of the *AR* and *INHA* genes. In addition, we show that *NR1H2*/LXRβ, a modulator of lipid and cholesterol homeostasis in the testis, regulates the *AR.* Variants in *NR5A1*/SF-1, *INHA*, and *AR* have been previously reported to cause monogenic DSD. Our study was not able to provide functional proof of the disease-causing effects of specific variants in *NR5A1*/SF-1, *INHA,* and *NR1H2*/LXRβ identified in combination in a 46,XY DSD individual. Addressing the possible oligogenic mode of these mechanisms remains a challenge.

## 4. Materials and Methods

### 4.1. Participants

The patient and his father included in this work were part of two previous genetic studies [9,27] and the SF1next study [5].

### 4.2. In Silico Analyses and Variant Classification

The DNA of the index patient and the father were sequenced by WES (Novogene, Cambridge, UK) and analyzed with an in-house specific data-filtering algorithm for gene variants related to DSD and/or *NR5A1*/SF-1 [9,11]. We predicted the possible effect of the identified genetic variants on the structure and function of the protein using Polyphen-2, (Polymorphism Phenotyping v2, http://genetics.bwh.harvard.edu/pph2/) (accessed on 7 February 2023), Panther (Protein ANalysis THrough Evolutionary Relationships, http://www.pantherdb.org/tools/csnpScore.do) (accessed on 7 February 2023), SNPs and Go (https://snps-and-go.biocomp.unibo.it/snps-and-go/) (accessed on 7 February 2023), CADD (Combined Annotation Dependent Depletion, https://cadd.gs.washington.edu/) (accessed on 7 February 2023), and the calibrated scores given by VarSome [28] for Revel (Rare Exome Variant Ensemble Learner), SIFT (scale-invariant feature transform), Provean (Protein Variation Effect Analyzer), Mutation Taster, and M-CAP (Mendelian Clinically Applicable Pathogenicity). Variants were classified for pathogenicity according to the standards and guidelines of the ACMG using VarSome version v.11.9.0 [28]. We explored the possible pathogenicity of multiple variants’ combinatory effects using ORVAL version 2.2.0 (Oligogenic Resource for Variant AnaLysis) [79].

### 4.3. Plasmids

The human HA-tagged WT and the variant c.58G>C cDNA of *NR5A1*/SF-1 (NM_004959.5) in pcDNA3, empty control vector pcDNA3, and *Renilla*-TK (pRL-*TK*) were all available from previous work [27]. The human *NR1H2* cDNA (NM_007121.5) in pCMV3-C-HA and *RXRA* cDNA (NM_002957.5) in the pCMV3 vector were purchased (Sino Biologicals Inc, Eschborn, Germany). The human *NR1H2* cDNA was used as a template to generate the *NR1H2* variant expression vector by PCR-based site-directed mutagenesis using the primers, forward (5′-CGGAAGAAGAAGATTCGGAACAAACAGCAGCAGGAG-3′), reverse (5′-CTCCTGCTGCTGTTTGTTCCGAATCTTCTTCTTCCG-3′), and the QuickChange protocol by Stratagene (Agilent Technologies Inc., Santa Clara, CA, USA).

### 4.4. Cloning

The 5′-untranslated region constructs of the different genes were produced by PCR using control human DNA extracted from blood leukocytes using the DNA isolation kit of Qiagen (QIAGEN, Aarhus, Denmark). The different forward primers used for PCR were as follows: −*2056_INHA* (5′-AGAGAGGGTACCTTGAGCACGAAGCCGCC-3′), −*520_INHA* (5′-AGA GAGGGTACCCTGAGGGGTGATGCACTTTGTC-3′), −*213_INHA* (5′-GAGGGTACCCA GACATCTGCGTCAGAGATAGGAG-3′), −*198_INHA* (5′-AGAGGGTACCGAGATAGGA GGTCTCAATGCCACG-3′). All had the KpnI restriction site included, while the following reverse primer, including the XhoI restriction site, was used in the four PCR reactions, (5′-GAGAGACTCGAGAGAACAAGTTCCCGGGCCAG-3′). For the generation of the −*220_GnRHR* construct, the forward primer including the KpnI restriction site (5′-AGAGGTACCGGCCTGCTCTGTTTTAGCACT-3′) and the reverse primer including the XhoI restriction site (5′-GAGCTCGAGATTTTCCCAGGACAGAGCTTCAAG-3′) were used. For the generation of the −*3000_AR* construct, the forward primer including the HindIII restriction site (5′-AGAGAAGCTTTAAACTTTGGAGTCTTTCAGACCCAG-3′), and the reverse primer including the XhoI restriction site (5′-GAGACTCGAGCCTTGAG CTTGGCTGAATCTTCC-3′) were used in the PCR reaction. All PCR products were digested with the indicated restriction enzymes and subcloned into the corresponding site in the pGL3 basic vector (Promega, Madison, WI, USA). The constructs were confirmed by direct sequencing. The −*2300_GnRHR* promoter construct in pGL3 was custom-made by Genscript (Rijswijk, the Netherlands).

### 4.5. In Vitro Testing of Transactivation Activity by Dual Luciferase Assay

Non-steroidogenic, human embryonic kidney HEK293T cells (ATCC CRL-1573), steroidogenic NCI-H295R adrenal cells (ATCC CRL-2128), and mouse Leydig MA-10 cells (ATCC CRL-3050) were cultured as previously described [27,80]. For all promoter activity experiments, cells were cultured on 12-well plates. For the *INHA* promoter activity experiments, NCI-H295R and MA-10 steroidogenic cells were transiently transfected with 950 ng from the different promoter luciferase reporter constructs, −*2050INHA*_pGL3, −*520INHA*_pGL3, −*213INHA*_pGL3, or −*198INHA*_pGL3; whereas HEK293T cells were transiently transfected with 200 ng WT or mutant *NR5A1*/SF-1 expression vectors, 800 ng of the different promoter luciferase reporter construct −*2050INHA*_pGL3, −*520INHA*_pGL3, −*213INHA*_pGL3, or −*198INHA*_pGL3 separately. For the *GnRHR* promoter activity experiment, HEK293T cells were transiently co-transfected with 200 ng WT or mutant *NR5A1*/SF-1 expression vectors and 800 ng of the promoter luciferase reporter constructs −*2300GnRHR*_pGL3 or −*220GnRHR*_pGL3. For the *AR* promoter activity experiments, MA-10 or NCI-H295R cells were transiently transfected with 950 ng of the −*3000AR*_pGL3 promoter luciferase reporter construct, while for the *AR* promoter experiments with overexpressed *NR5A1*/SF-1 or *NR1H2*/LXRβ, the cell lines were transiently transfected with 600 ng of the −*3000AR*_pGL3 promoter and 200 ng WT or mutant of *NR5A1*/SF-1 expression vector (in the three cell lines), or with *NR1H2*/LXRβ and RXRA expression vectors. Lastly, 50 ng of the pRL-TK vector was used as an internal control in all transfection experiments. All transfections were carried out with Lipofectamine 2000^TM^ (Invitrogen, Glasgow, UK) in Opti-MEM (1X)-reduced serum medium (Gibco, Thermo Fisher Scientific, Waltham, MA, USA). Forty-eight hours after transfection, the cells were washed with PBS, lysed, and assayed for luciferase activity with a Dual-Luciferase assay using a microplate Luminometer reader (Fluoroskan Ascent^®^ FL and Fluoroskan Ascent^®^, Thermo Fisher, Waltham, MA, USA). Specific *Firefly* luciferase readings were standardized against *Renilla* luciferase control readings. Experiments were repeated three to five times in duplicates, and the data were summarized, giving the mean ± standard error of the mean (SEM). Statistical significance was examined by Student’s *t*-test (GraphPad Prism, GraphPad Software version 9.4.1.681, Boston, MA, USA). Significance was assumed with a *p*-value of less than 0.05.

## Figures and Tables

**Table 1:** Biochemical characterization of the index patient at 15 years of age.

Hormones/Markers	Biochemical Value	Range	Units
Sex hormones			
FSH	**85.1**	0.95–11.95	mU/mL
LH	**20.3**	0.57–12.07	mU/mL
Prolactin	**29.1**	3.46–19.4	ng/mL
Testosterone	4.15	1–12	ng/mL
AMH	**5.18**	27–1141	pM
Adrenal function			
ACTH	**53.7**	9.0–40.0	pg/mL
Cortisol	179	30–210	ng/mL
DHEA-S	2243	166–2427	ng/mL

ACTH, adrenocorticotropic hormone; AMH, anti-Müllerian hormone; DHEA-S, dehydroepiandrosterone sulfate; FSH, follicle-stimulating hormone; LH, luteinizing hormone. Values in bold are outside the range.

**Table 2:** Genetic characterization of the different gene variants identified in a complex DSD case.

Gene Name	Gene Transcript	Variant	Chromosome Position	Type/ Consequence	ACMG Classification (Criteria)	SIFT	Polyphen	Mutation Taster	Panther	SNPs and Go	M-CAP	Mutation Assessor	REVEL	Provean	CADD Score	ORVAL—VarCoPP Score
*NR5A1*	ENST00000373588.9	c.58G>C; p.(Val20Leu)	9:124503338	SNV/missense	P	Unc	B	Unc	Prdam	Dis	P	Unc	P	Unc	23.3	ND
*NR1H2*	ENST00000253727.10	c.515_516insCAA; p.(Arg171_Lys172insAsn)	19:50378563	Ins/In-frame insertion	VUS	ND	ND	ND	ND	ND	ND	ND	ND	ND	ND	ND
*INHA*	ENST00000243786.3	c.675T>G; p.(Ser225Arg)	2:219575100	SNV/missense	B	B	B	B	ND	Dis	Unc	Unc	B	B	15.4	0.5825
*TCF7L2*	ENST00000355995.9	c.1535C>G; p.(Pro512Arg)	10:113165647	SNV/missense	LB	B	B	Unc	Prben	Neu	Unc	B	B	B	23.5	0.9825
*NIBAN1*	ENST00000367511.4	c.929G>A; p.(Arg310His)	1:184823223	SNV/missense	VUS	Unc	Prdam	Unc	Prdam	Neu	B	Unc	B	Unc	25.2	0.8500
*SCUBE2*	ENST00000649792.2	c.692C>T; p.(Thr231Ile)	11:9066765	SNV/missense	VUS	Unc	Prdam	Unc	Prdam	Neu	B	Unc	Unc	P	24.6	0.8450

ACMG, American College of Medical Genetics and Genomics; B, benign; Dis, disease-causing; DSD, disorder of sex development; Ins, insertion; LB, likely benign; ND, not defined; Neu, neutral; P, pathogenic; Prben, probably benign; Prdam, probably damaging; SNV, single-nucleotide variant; Unc, uncertain; VUS, variant of uncertain significance.

**Table 3:** Relevant information on selected candidate genes from literature.

Gene/Protein	Biological Function	Phenotype Associated with This Gene in Humans	The Phenotype Associated with This Gene in Mice Models	In Vitro Studies (*NR5A1*-Related)	A Possible Contribution of This Gene to the DSD Phenotype of the Patient?
*NR5A1*/SF-1	Necessary in the formation of the bipotential gonad; plays an important role in the expression of male-specific genes and participates in the ovarian development [29]. Main regulator of enzymes involved in adrenal and gonadal steroidogenesis [29]. Plays physiological roles in the central nervous system [29].	*NR5A1* homozygous and heterozygous variants are associated with disorders of sex development including adrenal insufficiency and 46,XY gonadal dysgenesis, ambiguous genitalia, hypospadias, micropenis, spermatogenic failure with normal genitalia, and primary ovarian insufficiency [29,30].	XY mice lacking *Nr5a1* have gonadal dysgenesis, adrenal insufficiency, and underdevelopment of the spleen [3]. *Nr5a1*−/− mice do not express luteinizing hormone or follicle-stimulating hormone and have a disorganized ventromedial nucleus of the hypothalamus [31].	The majority of heterozygous *NR5A1*/SF-1 variants located in the DNA-binding domain present with impaired functional activity on different human steroidogenic enzyme promoters, while variants located elsewhere in the SF-1 protein present with variable activity. Mostly, no genotype-phenotype correlation was found [6].	Yes
*NR1H2*/LXRβ	Plays an important role as a modulator of lipid homeostasis and inflammation throughout the human body [32].	Diseases associated with *NR1H2* include type 2 diabetes and male infertility (azoospermia) [32,33,34,35,36].	*Nr1h2*−/− mice are glucose-intolerant due to impaired insulin secretion, with a lost ability to regulate cholesterol, lipids, and carbohydrates, and with a defective immune function [36]. *Nr1h2*−/− mice have an excessive cholesterol accumulation in Sertoli cells from 2.5 months and dysregulated spermatogenesis at the age of 10 months [33].	LXRβ is involved in the basal expression levels of *CYP11A1*, *StAR*, and *NR5A1* in NCI-H295R adrenal cells [34].	Yes
*INHA*/Inhibin α	Antagonizes activin signaling in the reproductive hypothalamic-pituitary gonadal axis [37,38].	Homozygous *INHA* variants are associated with decreased prenatal and postnatal testosterone production and infertility in males, and primary ovarian failure in women [38,39,40].	*INHA* knockout mice develop mixed or incompletely differentiated gonadal stromal tumors and die from cachexia syndrome [38,41].	Rat inhibin alpha gene expression is regulated by the synergistic activity of *Nr5a1* and cAMP [42].	Yes
*TCF7L2*/TCF-4	Plays a role in intestinal cancer through the WNT signaling pathway [43,44]. Involved in the development of the small intestinal and colonic epithelium tissue homeostasis in the adult intestine [45,46].	*TCF7L2* variants are associated with an increased risk of type 2 diabetes [47,48,49].	*Tcf7l2* knockout causes neonatal death in mice [43]. Conditional inactivation of *Tcf7l2* in the adult intestinal epithelium in mice causes impaired cell proliferation in the small intestines and colon [46].	Tcf-4 is involved in rat inhibin alpha gene expression: Tcf-4 disrupts β-catenin’s ability to synergize with Sf-1 on the inhibin alpha promoter in a dose-dependent manner [50].	Unlikely
*NIBAN1*/FAM129A	Plays an important role in apoptosis, preventing cell death and tumor progression under stress conditions [51,52].	*NIBAN1* expression has been described in several tumor subtypes, including microcarcinomas, papillary and follicular carcinoma, and prostate cancer, as well as in Hashimoto’s Thyroiditis [51].	*Niban1*−/− mice are viable and show no obvious phenotype or any phenotypic abnormalities [52].	Not found	Unlikely
*SCUBE2*/SCUB2	Plays an important role as a tumor suppressor in different types of cancer [53,54].	*SCUBE2* expression is reduced in endometrial, breast, and colorectal cancers [53].	*Scube2*(−/−) mice have a defective endochondral bone formation and impaired Indian hedgehog-dependent chondrocyte-mediated chondrocyte differentiation and proliferation [55].	Not found	Unlikely

## Data Availability

Data were collected in a project-specific REDCap database governed by the Clinical Trials Unit (CTU) at the University of Bern, Switzerland. The genetic data are also stored on the servers of the University of Bern. These data can also be accessed upon reasonable request according to ethical considerations and informed consent.

## Supplementary Materials

The following supporting information can be downloaded at: https://www.mdpi.com/article/10.3390/ijms251810109/s1. References [81,82,83,84,85,86,87,88,89,90,91,92,93,94] are cited in the Supplementary Materials.
