# Supplementary material for: NR5A1/SF-1 Collaborates with Inhibin α and the Androgen Receptor
Source: Int J Mol Sci. 2024 Sep 20;25(18):10109. doi: 10.3390/ijms251810109 (PMC11432463; doi:10.3390/ijms251810109)
Supplement: Supplementary file 1 [file ijms-25-10109-s001.zip › ijms-3175587-supplementary.pdf]

International Journal of
Molecular Sciences

MDPI

Supplementary Materials

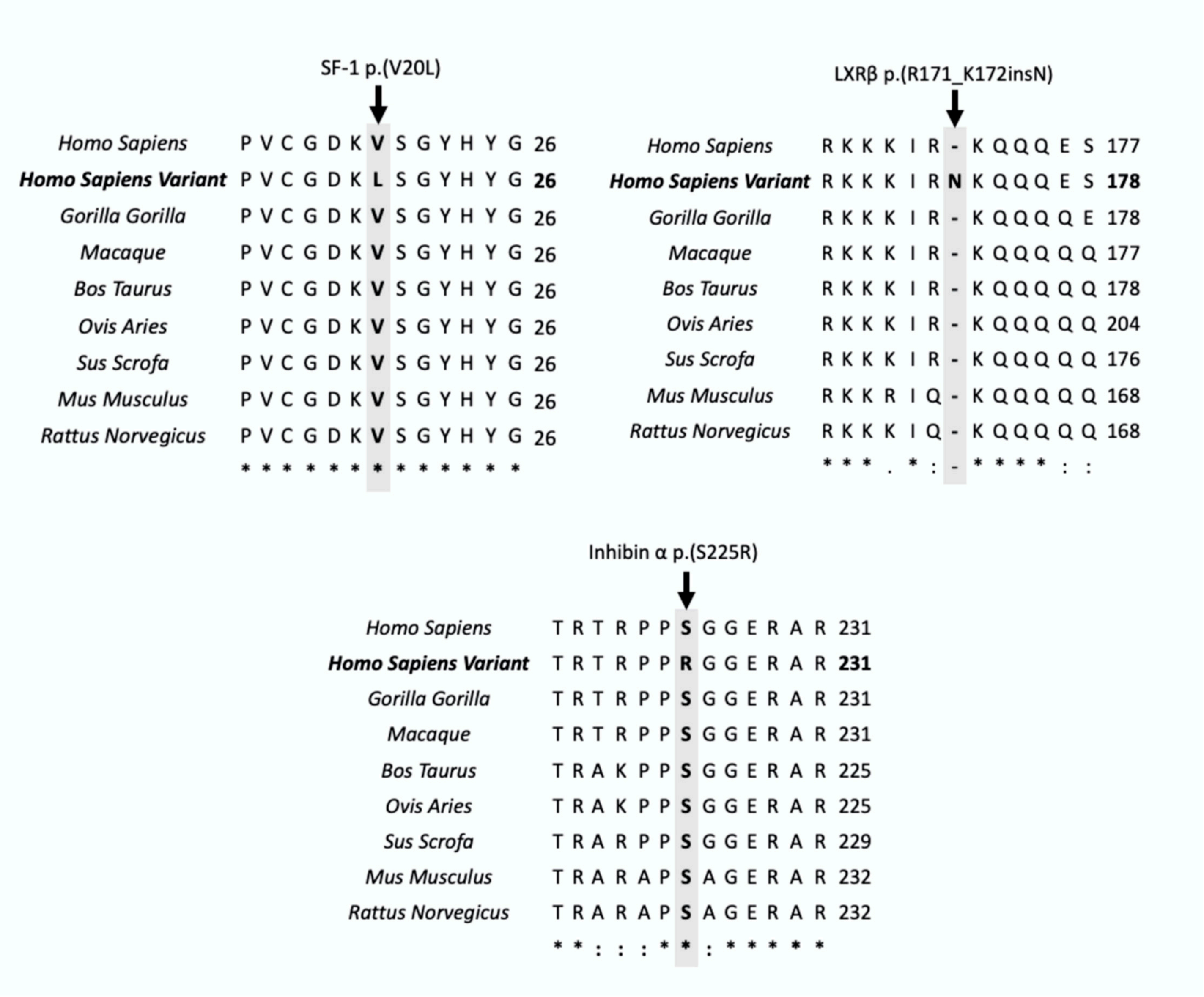

Figure S1: Protein localization and conservation across species of the three variants *NR5A1*/SF-1 p.(V20L), *NR1H2*/LXRβ p.(R171_K172insN) and *INHA*/inhibin α p.(S225R). The alignment of the various proteins across different species is shown. Localization of the identified human amino acid variants is indicated with arrows and seems highly conserved across species. *, conserved amino acid across species. :, different amino acid across species.

International Journal of
Molecular Sciences

MDPI

Table S1: Reported combined variants in *NR5A1* and associated genes

The DSD phenotype, *NR5A1* and *NR5A1*-associated gene variants, zygosity and the genetic test used for the variants identification are presented.

Patient phenotype*	*NR5A1*/SF-1 variant (NM_004959.5)	Genetic test	Name of the additional gene variant/ (transcript)	Gene variant	References
46,XY DSD	c.1256T>A[1]; p.(L419Q)	Panel	*AR* (NM_000044.6)	c.2659A>G[2]; p.(M887V)	[1]
46,XY DSD Hypospadias	c.251G>A[1]; p.(R84H)	Panel	*ZFPM2* (NM_012082.4)	c.2107A>C[1]; p.(M703L)	[1]
46,XY DSD CGD	c.630-636del[1]; p.(Y211Tfs*83)	WES	*TBX2* (NM_005994.4)	c.641A>G[1]; p.(N214S)	[2]
46,XY GD	c.937C>T[1]; p.(R313C)	Panel	*MAP3K1* (NM_005921.2)	c.710A>G[1]; p.(Q237R)	[3, 4]
46,XY DSD Micropenis, hypospadias	c.104G>T[1]; p.(G35V)	Panel	*BMP2* (NM_001200.4)	c.316G>A[1]; p.(A106T)	[4, 5]
46,XY DSD Vulvar dysplasia, no inguinal gonad	c.634G>A[1]; p.(G212S)	Panel	*SRY* (NM_003140.3)	c.227G>T[2]; p.(R76L)	[5]
			FGF10 (NM_004465.2)	c.610A>G[1]; p.(M204V)	[5]
46,XY DSD Micropenis, hypospadias, cryptorchidism	c.86C>A[1]; p.(T29K)	Panel	*AR* (NM_000044.6)	c.884T>C[1]; p.(L295P)	[5]
			MYH6 (NM_002471.4)	c.4231G>A[2]; p.(A1411T)	[5]
46,XY DSD, Micropenis, hypospadias, unilateral cryptorchidism	c.1109G>A[1]; p.(C370Y)	Panel	*SOX3* (NM_005634.3)	c.157G>C[1]; p.(V53L)	[5]
46,XY DSD Micropenis, hypospadias, cryptorchidism, gonadal dysplasia	c.1289G>T[1]; p.(S430I)	Panel	*HSD17B3* (NM_000197.2)	c.179T>C[1]; p.(I60T)	[5]
			WT1 (NM_001198551.2)	c.19A>C[1]; p.(T7P)	[5]
46,XY DSD Micropenis, hypospadias, cryptorchidism	c.849C>A[1]; p.(C283*)	Panel	*CST9* (NM_001008693.3)	c.259C>T[1]; p.(R87*)	[5]

46,XY DSD, Micropenis, hypospadias, unilateral cryptorchidism	c.1100A>G[1]; p.(E367G)	Panel	*CST9* (NM_001008693.3)	c.259C>T[1]; p.(R87*)	[5]
46,XY DSD Micropenis, hypospadias	c.265del[1]; p.(R89fs*105)	Panel	*EGF* (NM_001178131.3)	c.2995G>A[1]; p.(V999M)	[5]
46,XY DSD	c.1171A>T[1]; p.(K391*)	Panel	*ATRX* (NM_000489.6)	c.2595C>G[1]; p.(H865Q)	[6]
			AMH (NM_000479.5)	c.-2C>T[1]	[6]
46,XY DSD GD	c.251G>A[1]; p.(R84H)	Panel	*ZFPM2* (NM_012082.4)	c.2107A>C[1]; p.(M703L)	[7, 8]
46,XY DSD Hypospadias	c.1114_1116del[1]; p.(K372del)	Panel	*SRD5A2* (NM_000348.4)	c.680G>A[1]; p.(R227Q)	[7, 9]
46,XY DSD, Ambiguous genitalia	c.70C>T[1]; p.(H24Y)	WES	*AKR1C3* (NM_003739.6)	c.548A>G[1]; p.(L183R)	[10]
			DOCK8 (NM_203447.4)	c.1139T>C[1]; p.(I380T)	[10]
			FSHR (NM_000145.4)	c.1532A>G[1]; p.(Y511C)	[10]
			NCOR1 (NM_006311.3)	c.6754C>T[1]; p.(H2252Y) & c.6544G>A[1]; p.(A2182T)	[10]
			POR (NM_001382662.3)	c.1264T>G[1]; p.(W422G)	[10]
46,XY DSD Bilateral inguinal hernia	c.70delC[1]; p.(H24Tfs*51)	WES	*CACNG4* (NM_014405.4)	c.715C>T[1]; p.(R239W)	[10, 11]
			FBLN2 (NM_001004019.2)	c.385G>A[1]; p.(D129N)	[10]
			NAV1 (NM_020443.5)	c.2947C>A[1]; p.(P983T)	[10]
			SMAD6 (NM_005585.5)	c.1455dupC[1]; p.(C486Lfs*79)	[10]
			SRA1 (NM_001035235.3)	c.94C>G[1]; p.(Q32E)	[10]
			ZDHHC11 (NM_024786.3)	c.676G>A[1]; p.(V226M)	[10]
			ZFPM2 (NM_012082.4)	c.302G>A[1]; p.(G101E)	[10]
46,XY DSD	c.614_615insC[1]; p.(Q206Tfs*20)	WES	*CHD7* (NM_017780.4)	c.7579A>C[1]; p.(M2527L)	[10, 11]

International Journal of Molecular Sciences

MDPI

Female external genitalia			*DENND1A* (NM_020946.2)	c.2351C>A[1]; p.(A784D)	[10]
			GDNF (NM_001190468.1)	c.328C>T[1]; p.(R110W)	[10]
			GLI2 (NM_001371271.1)	c.4333C>T[1]; p.(L1445F)	[10]
			SOX30 (NM_178424.2)	c.455C>T[1] ; p.(Pro152Leu)	[10]
46,XY DSD Hypospadias, cryptorchidism	c.58G>C[1]; p.(V20L)	WES	*INHA* (NM_002191.4)	c.675T>G[1]; p.(S225R)	[10, 11]
46,XY DSD	c.88T>A[1]; p.(C30S)	WES	*STAR* (NM_000349.3)	c.361C>T[1]; p.(R121W)	[12]
46,XY DSD	c.614_615insC[1]; p.(Q206Tfs*20)	WES	*AMH* (NM_000479.5)	c.428C>T[1]; p.(T143l)	[11, 12]
46,XY DSD	c.1183_1185delGAG [1]; p.(E395del)	WES	*ZFPM2* (NM_012082.4)	c.1632G>A[1]; p.(M544l)	[12, 13]
46,XY DSD PGD	c.288_304del[1]; p.(M98Gfs*45)	WES	*DHX37* (NM_032656.4)	c.1399C>G[1]; p.(L467V)	[14]
46,XY DSD PGD	c.11C>A[1]; p.(S4*)	WES	*DHX37* (NM_032656.4)	c.2995G>A[1]; p.(V999M)	[14]
46,XY DSD (CAIS)	Deletion of exon 7. (~32 kb affecting chr9:127213317-127245328)[1].	CNV Analysis	*AR*	Duplication of exon 2 of the X-linked *AR* gene	[15]
		CNV analysis	*FSHR*	Deletion of exons 2–10. (~105 kb region affecting chr2:49189845-49295452)[3]	[15]
46,XY DSD Micropenis	c.-762C>T[1]	WES	*SRA1* (NM_001035235.4)	c.478C>T[1]; p.(R160W)	[16]
46,XY PGD	c.-413G>A[1] and c.-207C>A[1]	WES	*WDR11* (NM_018117.12)	c.3033_3036del[1]; p.(D1011Efs*21)	[16]
			WWOX (NM_016373.4)	c.1088C>A[1]; p.(A363D)	[16]
46,XY DSD Ambiguous genitalia, GD	c.937C>T[1]; p.(R313C)	WES	*MAP3K1* (NM_005921.2)	c.710A>G[1]; p.(E237R)	[17]
46,XY DSD Hypospadias	c.929A>C[1]; p.(H310P)	WES	*MAP3K1* (NM_005921.2)	c.2282T>C[1]; p. (I761T)	[18]
46,XY DSD Hypospadias	c.991-1G>C[1]; p.(G330_S331del)	WES	*OTX2* (NM_021728.4)	c.425C>G[1]; p.(P134R)/p.(P142R)	[19]

46,XDY DSD Hypospadias	c.991-1G>C[1]; p.(G330_S331del)	WES	*PROP1* (NM_006261.5)	c.301_302delAG[1]; p.(L102CfsTer8)	[19]
46,XY Mild DSD	c.437G>C[1]; p.(G146A)	WES	*FGFR3* (NM_000142.5)	c.1633_1634del[1]; p.(C545Hfs*17)	[20]
			ADAMTS16 (NM_139056.4)	c.1822_1823del[1]; p.(H608*)	[20]
			INSR (NM_000208.4)	c.660_661del[1]; p.(P220Hfs*4)	[20]
46,XY Severe DSD	c.437G>C[1]; p.(G146A)	WES	*GLI2* (NM_001371271.1)	c.3528G>T[1]; p.(Q1176H)	[20]
			CHD7 (NM_017780.4)	c.1623C>A[1]; p.(H541Q)	[20]
			MYO7A (NM_000260.4)	c.2882G>A[1]; p.(G961A)	[20]
			VDR (NM_000376.3)	c.176C>T[1]; p.(T59I)	[20]
46,XY Mild DSD	c.437G>C[1]; p.(G146A)	WES	*NRP1* (NM_003873.7)	c.182C>A[1]; p.(P61Q)	[20]
46,XY Opposite sex DSD	c.437G>C[1]; p.(G146A)	WES	*LHCGR* (NM_000233.4)	c.757T>C[3]; p.(S253P)	[20]
46,XX Opposite sex DSD	c.437G>C[3]; p.(G146A)	WES	*COL27A1* (NM_032888.4)	c.3715C>T[1]; p.(R1239W)	[20]
			TYRO3 (NM_006293.4)	c.666_667insCACTGCCTGCAGCCCCCTTCAACATCACC[1]; p.(A223Hfs*21)	[20]
46,XY Mild DSD	c.437G>C[1]; p.(G146A)	WES	*SOX8* (NM_014587.5)	c.676A>C[1]; p.(T226P)	[20]
46,XY Severe DSD	c.437G>C[1]; p.(G146A)	WES	*POR* (NM_001395413.1)	c.1679C>T[1]; p.(T560M)	[20]
			PKD1 (NM_001009944.3)	c.2624C>T[1]; p.(P875L)	[20]
			SRCAP (NM_006662.3)	c.7142G>A[1]; p.(R2381H)	[20]
			SOX9 (NM_000346.4)	c.710dup[1]; p.(P238Tfs*14)	[20]
46,XY Opposite sex DSD	c.437G>C[1]; p.(G146A)	WES	*AR* (NM_000044.6)	c.2323C>T[2]; p.(R775C)	[20]

International Journal of Molecular Sciences MDPI

46,XY Idiopathic oligozoospermia	c.1063G>A[1]; p.(V355M)	Panel	*USP9Y* (NM_004654.4)	c.3178G>A[2]; p.(A1060T)	[21]
46,XX BPES and POI	c.578T>A[1]; p.(I193N)	Sanger sequencing	*LHX4* (NM_033343.4)	c.250C>T[1]; p.(R84C)	[22]
46,XX DSD	c.486C>T[n/a]; p.(=)	Panel	*LHCGR* (NM_000233.4)	c.458+3A>G[n/a]	[6]
46,XY DSD	c.77C>T[1]; p.(G26V)	Panel	*DHX37* (NM_032656.4)	c.1474G>C[1]; p.(A494P)	[23]
46,XX POI	c.1233C>T[1]; p.(H410=)	Panel	*FSHR* (NM_000145.4)	c.1664C>T[1]; p.(T555I)	[24]
46,XY DSD	c.634G>A[n/a]; p.(G212S)	Sanger sequencing	*SRY* (NM_003140.3)	c.227G>T[n/a]; p.(R76L)	[25]
			FGF10 (NM_004465.2)	c.610A>G[n/a]; p.(M204V)	[25]
46,XY DSD Micropenis	c.1223A>C[1]; p.(H408P)	WES	*MAP3K1* (NM_005921.2)	c.3418A>G[1]; p.(M1140V)	[26]
			CTU2 (NM_001012759.3)	c.710C>T[1]; p.(A237V)	[26]
47,XXY Opposite sex DSD	c.50G>T[1]; p.(G17V)	WES	*AMH* (NM_000479.5)	c.300C>T[1]; p.(F100=)	[27]
47,XY Severe DSD	c.218G>C[1]; p.(C73S)	WES	*SRD5A2* (NM_000348.4)	c.265C>G[3]; p.(L89V)	[27]
47,XY Severe DSD	c.219C>G[1]; p.(C73W)	WES	*SZT2* (NM_015284.4)	c.4039C>T[1]; p.(R1347C)	[27]
			ALMS1 (NM_015120.4)	c.4207A>G[1]; p.(T403A)	[27]
			BTD (NM_000060.4)	c.1330G>C[1]; p.(D444H)	[27]
			TREX1 (NM_033629.6)	c.206T>C[1]; p.(L69P)	[27]
			PDHB (NM_000925.4)	c.701-3C>T[1]	[27]
			VCAN (NM_004385.5)	c.1412C>T[1]; p.(T471M)	[27]
			SYNE1 (NM_033071.5)	c.14732G>A[1]; p.(R4911H)	[27]
			ARID1B (NM_020732.3)	c.369_392dup[1]; p.(Q124_Q131dup)	[27]

International Journal of *Molecular Sciences*

MDPI

			RELN (NM_005045.4)	c.865A>G[1]; p.(N289D)	[27]
			ARMC4 (NM_018076.5)	c.1386+5G>A[1]	[27]
			DYNC2H1 (NM_001377.3)	c.4295T>C[1]; p.(I1432T)	[27]
			HEPACAM (NM_152722.5)	c.139G>A[1]; p.(V47M)	[27]
			GALC (NM_000153.4)	c.1685T>C[1]; p.(I562T)	[27]
			OCA2 (NM_000275.3)	c.1025A>G[1]; p.(Y342C)	[27]
			GAA (NM_000152.5)	c.2065G>A[1]; p.(E689K)	[27]
			SIK1 (NM_173354.5)	c.1784G>A[1]; p.(R595Q)	[27]
46,XY Opposite sex DSD	c.680T>C[1]; p.(I227T)	WES	*POR* (NM_000941.3)	c.859G>C[1]; p.(A287P)	[27]

DSD, disorders/differences of sex development; CGD, complete gonadal dysgenesis; WES, whole-exome sequencing; GD,gonadal dysgenesis; PGD, primary gonadal dysgenesis; CAIS, complete androgen insensitivity; CNV, Copy number variations; BPES: blepharoptosis–ptosis–epicanthus syndrome; POI: primary ovarian insufficiency.[1] Heterozygous variant [2] Hemizygous variant; [3] Homozygous variant; n/a: not available. *The clinical phenotype is indicated when available and according to the description in the corresponding publication.

References

1. Eggers, S.; Sadedin, S.; van den Bergen, J. A.; Robevska, G.; Ohnesorg, T.; Hewitt, J.; Lambeth, L.; Bouty, A.; Knarston, I. M.; Tan, T. Y.; Cameron, F.; Werther, G.; Hutson, J.; O'Connell, M.; Grover, S. R.; Heloury, Y.; Zacharin, M.; Bergman, P.; Kimber, C.; Brown, J.; Webb, N.; Hunter, M. F.; Srinivasan, S.; Titmuss, A.; Verge, C. F.; Mowat, D.; Smith, G.; Smith, J.; Ewans, L.; Shalhoub, C.; Crock, P.; Cowell, C.; Leong, G. M.; Ono, M.; Lafferty, A. R.; Huynh, T.; Visser, U.; Choong, C. S.; McKenzie, F.; Pachter, N.; Thompson, E. M.; Couper, J.; Baxendale, A.; Gecz, J.; Wheeler, B. J.; Jefferies, C.; MacKenzie, K.; Hofman, P.; Carter, P.; King, R. I.; Krausz, C.; van Ravenswaaij-Arts, C. M.; Looijenga, L.; Drop, S.; Riedl, S.; Cools, M.; Dawson, A.; Juniarto, A. Z.; Khadilkar, V.; Khadilkar, A.; Bhatia, V.; Dũng, V. C.; Atta, I.; Raza, J.; Thi Diem Chi, N.; Hao, T. K.; Harley, V.; Koopman, P.; Warne, G.; Faradz, S.; Oshlack, A.; Ayers, K. L.; Sinclair, A. H., Disorders of sex development: insights from targeted gene sequencing of a large international patient cohort. *Genome Biol* **2016,** 17, (1), 243.
2. Werner, R.; Mönig, I.; Lünstedt, R.; Wünsch, L.; Thorns, C.; Reiz, B.; Krause, A.; Schwab, K. O.; Binder, G.; Holterhus, P.-M.; Hiort, O., New NR5A1 mutations and phenotypic variations of gonadal dysgenesis. *PLoS One* **2017,** 12, (5), e0176720-e0176720.
3. Mazen, I.; Abdel-Hamid, M.; Mekkawy, M.; Bignon-Topalovic, J.; Boudjenah, R.; El Gammal, M.; Essawi, M.; Bashamboo, A.; McElreavey, K., Identification of NR5A1 Mutations and Possible Digenic Inheritance in 46,XY Gonadal Dysgenesis. *Sex Dev* **2016,** 10, (3), 147-51.

4. Allali, S.; Muller, J.-B.; Brauner, R.; Lourenço, D.; Boudjenah, R.; Karageorgou, V.; Trivin, C.; Lottmann, H.; Lortat-Jacob, S.; Nihoul-Fékété, C.; De Dreuzy, O.; McElreavey, K.; Bashamboo, A., Mutation Analysis of NR5A1 Encoding Steroidogenic Factor 1 in 77 Patients with 46, XY Disorders of Sex Development (DSD) Including Hypospadias. *PLoS One* **2011,** 6, (10), e24117.
5. Wang, H.; Zhang, L.; Wang, N.; Zhu, H.; Han, B.; Sun, F.; Yao, H.; Zhang, Q.; Zhu, W.; Cheng, T.; Cheng, K.; Liu, Y.; Zhao, S.; Song, H.; Qiao, J., Next-generation sequencing reveals genetic landscape in 46, XY disorders of sexual development patients with variable phenotypes. *Human Genetics* **2018,** 137, (3), 265-277.
6. Hughes, L. A.; McKay-Bounford, K.; Webb, E. A.; Dasani, P.; Clokie, S.; Chandran, H.; McCarthy, L.; Mohamed, Z.; Kirk, J. M. W.; Krone, N. P.; Allen, S.; Cole, T. R. P., Next generation sequencing (NGS) to improve the diagnosis and management of patients with disorders of sex development (DSD). *Endocr Connect* **2019,** 8, (2), 100-110.
7. Robevska, G.; van den Bergen, J. A.; Ohnesorg, T.; Eggers, S.; Hanna, C.; Hersmus, R.; Thompson, E. M.; Baxendale, A.; Verge, C. F.; Lafferty, A. R.; Marzuki, N. S.; Santosa, A.; Listyasari, N. A.; Riedl, S.; Warne, G.; Looijenga, L.; Faradz, S.; Ayers, K. L.; Sinclair, A. H., Functional characterization of novel NR5A1 variants reveals multiple complex roles in disorders of sex development. *Hum Mutat* **2018,** 39, (1), 124-139.
8. Köhler, B.; Lin, L.; Ferraz-de-Souza, B.; Wieacker, P.; Heidemann, P.; Schröder, V.; Biebermann, H.; Schnabel, D.; Grüters, A.; Achermann, J. C., Five novel mutations in steroidogenic factor 1 (SF1, NR5A1) in 46,XY patients with severe underandrogenization but without adrenal insufficiency. *Hum Mutat* **2008,** 29, (1), 59-64.
9. Eggers, S.; Smith, K. R.; Bahlo, M.; Looijenga, L. H. J.; Drop, S. L. S.; Juniarto, Z. A.; Harley, V. R.; Koopman, P.; Faradz, S. M. H.; Sinclair, A. H., Whole exome sequencing combined with linkage analysis identifies a novel 3 bp deletion in NR5A1. *Eur J Hum Genet* **2015,** 23, (4), 486-493.
10. Camats, N.; Fernández-Cancio, M.; Audí, L.; Schaller, A.; Flück, C. E., Broad phenotypes in heterozygous NR5A1 46,XY patients with a disorder of sex development: an oligogenic origin? *Eur J Hum Genet* **2018,** 26, (9), 1329-1338.
11. Camats, N.; Pandey, A. V.; Fernandez-Cancio, M.; Andaluz, P.; Janner, M.; Toran, N.; Moreno, F.; Bereket, A.; Akcay, T.; Garcia-Garcia, E.; Munoz, M. T.; Gracia, R.; Nistal, M.; Castano, L.; Mullis, P. E.; Carrascosa, A.; Audi, L.; Fluck, C. E., Ten novel mutations in the NR5A1 gene cause disordered sex development in 46,XY and ovarian insufficiency in 46,XX individuals. *J Clin Endocrinol Metab* **2012,** 97, (7), E1294-306.
12. Martinez de LaPiscina, I.; Mahmoud, R. A.; Sauter, K. S.; Esteva, I.; Alonso, M.; Costa, I.; Rial-Rodriguez, J. M.; Rodriguez-Estevez, A.; Vela, A.; Castano, L.; Fluck, C. E., Variants of STAR, AMH and ZFPM2/FOG2 May Contribute towards the Broad Phenotype Observed in 46,XY DSD Patients with Heterozygous Variants of NR5A1. *Int J Mol Sci* **2020,** 21, (22).
13. Bashamboo, A.; Brauner, R.; Bignon-Topalovic, J.; Lortat-Jacob, S.; Karageorgou, V.; Lourenco, D.; Guffanti, A.; McElreavey, K., Mutations in the FOG2/ZFPM2 gene are associated with anomalies of human testis determination. *Hum Mol Genet* **2014,** 23, (14), 3657-65.
14. de Oliveira, F. R.; Mazzola, T. N.; de Mello, M. P.; Francese-Santos, A. P.; Lemos-Marini, S. H. V.; Maciel-Guerra, A. T.; Hiort, O.; Werner, R.; Guerra-Junior, G.; Fabbri-Scallet, H., DHX37 and NR5A1 Variants Identified in Patients with 46,XY Partial Gonadal Dysgenesis. *Life (Basel)* **2023,** 13, (5).
15. Sreenivasan, R.; Bell, K.; van den Bergen, J.; Robevska, G.; Belluoccio, D.; Dahiya, R.; Leong, G. M.; Dulon, J.; Touraine, P.; Tucker, E. J.; Ayers, K.; Sinclair, A., Whole exome sequencing reveals copy number variants in individuals with disorders of sex development. *Mol Cell Endocrinol* **2022,** 546, 111570.
16. Fabbri-Scallet, H.; Werner, R.; Guaragna, M. S.; de Andrade, J. G. R.; Maciel-Guerra, A. T.; Hornig, N. C.; Hiort, O.; Guerra-Junior, G.; de Mello, M. P., Can Non-Coding NR5A1 Gene Variants Explain Phenotypes of Disorders of Sex Development? *Sex Dev* **2022,** 16, (4), 252-260.
17. Mazen, I.; Mekkawy, M.; Kamel, A.; Essawi, M.; Hassan, H.; Abdel-Hamid, M.; Amr, K.; Soliman, H.; El-Ruby, M.; Torky, A.; El Gammal, M.; Elaidy, A.; Bashamboo, A.; McElreavey, K., Advances in genomic diagnosis of a large cohort of Egyptian patients with disorders of sex development. *Am J Med Genet A* **2021,** 185, (6), 1666-1677.
18. Cheng, Y.; Chen, J.; Zhou, X.; Yang, J.; Ji, Y.; Xu, C., Characteristics and possible mechanisms of 46, XY differences in sex development caused by novel compound variants in NR5A1 and MAP3K1. *Orphanet J Rare Dis* **2021,** 16, (1), 268.
19. Laan, M.; Kasak, L.; Timinskas, K.; Grigorova, M.; Venclovas, C.; Renaux, A.; Lenaerts, T.; Punab, M., NR5A1 c.991-1G > C splice-site variant causes familial 46,XY partial gonadal dysgenesis with incomplete penetrance. *Clin Endocrinol (Oxf)* **2021,** 94, (4), 656-666.
20. Martinez de Lapiscina, I.; Kouri, C.; Aurrekoetxea, J.; Sanchez, M.; Naamneh Elzenaty, R.; Sauter, K. S.; Camats, N.; Grau, G.; Rica, I.; Rodriguez, A.; Vela, A.; Cortazar, A.; Alonso-Cerezo, M. C.; Bahillo, P.; Bertholt, L.; Esteva, I.; Castano, L.; Fluck, C. E., Genetic reanalysis of patients with a difference of sex development carrying the NR5A1/SF-1 variant p.Gly146Ala has discovered other likely disease-causing variations. *PLoS One* **2023,** 18, (7), e0287515.
21. Cannarella, R.; Condorelli, R. A.; Paolacci, S.; Barbagallo, F.; Guerri, G.; Bertelli, M.; La Vignera, S.; Calogero, A. E., Next-generation sequencing: toward an increase in the diagnostic yield in patients with apparently idiopathic spermatogenic failure. *Asian J Androl* **2021,** 23, (1), 24-29.
22. Giannakopoulos, A.; Sertedaki, A.; Chrysis, D., A human paradigm of LHX4 and NR5A1 developmental gene interaction in the pituitary gland and ovary? *Eur J Hum Genet* **2022,** 30, (10), 1191-1194.

23. Gomes, N. L.; Batista, R. L.; Nishi, M. Y.; Lerario, A. M.; Silva, T. E.; de Moraes Narcizo, A.; Benedetti, A. F. F.; de Assis Funari, M. F.; Faria Junior, J. A.; Moraes, D. R.; Quintao, L. M. L.; Montenegro, L. R.; Ferrari, M. T. M.; Jorge, A. A.; Arnhold, I. J. P.; Costa, E. M. F.; Domenice, S.; Mendonca, B. B., Contribution of Clinical and Genetic Approaches for Diagnosing 209 Index Cases With 46,XY Differences of Sex Development. *J Clin Endocrinol Metab* **2022,** 107, (5), e1797-e1806.
24. Oral, E.; Toksoy, G.; Sofiyeva, N.; Celik, H. G.; Karaman, B.; Basaran, S.; Azami, A.; Uyguner, Z. O., Clinical and Genetic Investigation of Premature Ovarian Insufficiency Cases from Turkey. *J Gynecol Obstet Hum Reprod* **2019,** 48, (10), 817-823.
25. Wang, N.; Zhu, W.; Han, B.; Wang, H.; Zhu, H.; Chen, Y.; Chen, Y.; Liu, J.; Liu, Y.; Zhao, S.; Song, H.; Qiao, J., Inherited Missense Mutation Occurring in Arginine76 of the SRY Gene Does Not Account for Familial 46, XY Sex Reversal. *J Clin Endocrinol Metab* **2020,** 105, (5).
26. Zidoune, H.; Ladjouze, A.; Chellat-Rezgoune, D.; Boukri, A.; Dib, S. A.; Nouri, N.; Tebibel, M.; Sifi, K.; Abadi, N.; Satta, D.; Benelmadani, Y.; Bignon-Topalovic, J.; El-Zaiat-Munsch, M.; Bashamboo, A.; McElreavey, K., Novel Genomic Variants, Atypical Phenotypes and Evidence of a Digenic/Oligogenic Contribution to Disorders/Differences of Sex Development in a Large North African Cohort. *Front Genet* **2022,** 13, 900574.
27. Naamneh Elzenaty, R.; Martinez de Lapiscina, I.; Kouri, C.; Sauter, K. S.; Sommer, G.; Castano, L.; Fluck, C. E.; group, S. F. n. s., Characterization of 35 novel NR5A1/SF-1 variants identified in individuals with atypical sexual development: The SF1next study. *J Clin Endocrinol Metab* **2024**.
